
JOURNAL INFORMATION
==============================
NLM Title Abbreviation: Inter Econ
Journal ID: Inter Econ
NLM Journal ID: 101086399

Inter Economics

ISSN: 0020-5346

ARTICLE INFORMATION
==============================
PMCID: PMC7276106
DOI: 10.1007/s10272-020-0884-2
Article ID: 884
Article version: 1
Subjects: Forum Introduction

The European Response to the Coronavirus Crisis

Electronic publication date: 2020 Jun 7
Print publication date: 2020
Volume: 55
Issue: 3
First page: 132
Last page: 132
Copyright: © The Author(s) 2020
License: Open Access This article is licensed under a Creative Commons Attribution 4.0 International License, which permits use, sharing, adaptation, distribution and reproduction in any medium or format, as long as you give appropriate credit to the original author(s) and the source, provide a link to the Creative Commons licence, and indicate if changes were made.
The images or other third party material in this article are included in the article’s Creative Commons licence, unless indicated otherwise in a credit line to the material. If material is not included in the article’s Creative Commons licence and your intended use is not permitted by statutory regulation or exceeds the permitted use, you will need to obtain permission directly from the copyright holder.
To view a copy of this licence, visit http://creativecommons.org/licenses/by/4.0/.
License URL: https://creativecommons.org/licenses/by/4.0/

issue-copyright-statement: © The Author(s) 2020

==============================
DOI: 10.1007/s10272-020-0884-2

### The European Response to the Coronavirus Crisis

**On 11 March 2020, the World Health Organization declared the novel coronavirus outbreak a global pandemic. Ill-prepared and reluctant to halt economic activities, EU member states were slow to react to contain the spread of the virus. Although necessary, the lockdown measures came with a heavy price. The need for economic and financial assistance for EU member states with limited fiscal space has never been more pressing. The response to the economic damage this crisis has inflicted on the Union must be joint and swift – the future of the EU depends on it.**

### The Need for Monetary Financing of Corona Budget Deficits

**Paul de Grauwe**, London School of Economics, UK.

### How the COVID-19 Pandemic Is Reshaping the Trade Landscape and What to Do About It

**Sébastien Jean**, Centre d'Études Prospectives et d'Informations Internationales; and National Research Institute for Agriculture, Food and Environment (INRAE), Paris, France.

### SURE – EU Capacity for Stabilising Employment and Incomes in the Pandemic

**László Andor**, Foundation for European Progressive Studies, Brussels, Belgium.

### The Privilege of Working from Home at the Time of Social Distancing

**Armanda Cetrulo**, Scuola Superiore Sant'Anna, Pisa, Italy.

**Dario Guaracio**, Sapienza University of Rome, Italy.

**Maria Enrica Virgillito**, Scuola Superiore Sant'Anna, Pisa, Italy.

### Decades of Tight Fiscal Policy Have Left the Health Care System in Italy Ill Prepared to Fight the COVID-19 Outbreak

**Franz Prante**, Berlin School of Economics and Law (HWR), Germany.

**Alessandro Bramucci**, Berlin School of Economics and Law (HWR), Germany.

**Achim Truger**, German Council of Economic Experts, Wiesbaden; and University of Duisburg-Essen, Germany.

### Why Current EU Proposals for Corona-Related Financial Aid Cannot Replace Coronabonds

**Sebastian Dullien**, Macroeconomic Policy Institute (IMK), Düsseldorf, and HTW Berlin – University of Applied Sciences, Germany.

**Thomas Theobald**, University of Bamberg; and Macroeconomic Policy Institute (IMK), Düsseldorf, Germany.

**Silke Tober**, Macroeconomic Policy Institute (IMK), Düsseldorf, Germany.

**Andrew Watt**, Macroeconomic Policy Institute (IMK), Düsseldorf, Germany.

### Whither Coronabonds? The Past and Future of the EMU in the Coronavirus Pandemic 2020

**Bodo Herzog**, ESB Business School, Reutlingen, Germany.

### On the Macroeconomic and Social Impact of the Coronavirus Pandemic in Latin America and the Developing World

**Christian R. Proaño**, University of Bamberg, Germany.

### Common Pitfalls in the Interpretation of COVID-19 Data and Statistics

**Andreas Backhaus**, Federal Institute for Population Research, Wiesbaden, Germany.
